# Mixed-Solvent-Regulated MOF-Derived Porous In_2_O_3_ Nanostructures for Enhanced Triethylamine Gas Sensing

**DOI:** 10.3390/ma19163442

**Published:** 2026-08-13

**Authors:** Shuhao Shen, Jing Li, Rui Fang, Yongli Zhu, Wenbo Qin

**Affiliations:** School of Information Engineering, Suzhou University, Suzhou 234000, China; shenshuhao@ahszu.edu.cn (S.S.); lj@stu.ahszu.edu.cn (J.L.); fr@stu.ahszu.edu.cn (R.F.); zyl@stu.ahszu.edu.cn (Y.Z.)

**Keywords:** MOF, In_2_O_3_, mixed solvent, TEA, gas sensor

## Abstract

**Highlights:**

- Mixed solvents regulate MOF-derived porous In_2_O_3_ nanostructures.
- MOF-In_2_O_3_-2 shows the best TEA sensing response at 240 °C.
- High porosity and oxygen vacancies enhance TEA adsorption and reaction.

**Abstract:**

The detection of triethylamine (TEA) at low concentrations requires sensing materials with high surface reactivity and efficient gas-transport capability. In this work, porous In_2_O_3_ nanostructures were successfully prepared through a mixed-solvent-regulated metal–organic framework-derived (MOF) strategy. Indium nitrate and terephthalic acid were used as the metal source and organic ligand, respectively. By adjusting the volume ratio of N,N-dimethylformamide and ethanol, the nucleation and growth of In-based MOF precursors were effectively regulated, followed by thermal conversion into porous MOF-derived In_2_O_3_ materials. Structural characterization confirms that all samples were completely transformed into cubic In_2_O_3_ after calcination and exhibited porous architectures assembled from In_2_O_3_ nanoparticles. The solvent composition was found to exert a pronounced influence on the pore structure, defect concentration, and surface oxygen species. Gas-sensing measurements reveal that the MOF-In_2_O_3_ sensor delivered the best TEA-sensing performance at 240 °C, with a response of 77 toward 100 ppm TEA, relatively fast response/recovery behavior, a detection limit down to 0.5 ppm, and good selectivity and long-term stability. The superior performance can be attributed to the continuous gas-diffusion channels constructed by nanoparticle assembly, abundant oxygen vacancies and chemisorbed oxygen species that promote surface oxidation reactions, and the effective catalytic oxidation capability of In_2_O_3_ toward TEA molecules. This study demonstrates that regulating the solvent composition during MOF precursor synthesis is a simple and effective route to optimize the microstructure and surface defects of In_2_O_3_ for improved TEA gas sensing.

## 1. Introduction

Triethylamine (TEA) is a representative volatile organic amine that is widely used in organic synthesis, pharmaceuticals, pesticides, dyes, resins, and other chemical industries. However, it has a strong irritating odor and certain toxicity. Long-term exposure to TEA may cause adverse effects on the respiratory tract, eyes, and skin [1,2,3]. In addition, TEA is regarded as an important volatile marker generated from the decomposition of nitrogen-containing organic compounds during food spoilage, especially in protein-rich products [4,5]. Consequently, the advancement of gas sensors capable of rapid, sensitive, and selective detection of triethylamine (TEA) is of considerable significance.

Metal oxide semiconductor (MOS) gas sensors have garnered extensive attention in the detection of volatile organic compounds due to their simple device structure, low cost, high sensitivity, and compatibility with miniaturized and integrated sensing systems [6,7]. Among various metal oxides, In_2_O_3_ is a typical n-type wide-bandgap semiconductor with high electron mobility, good chemical stability, and strong surface oxygen adsorption ability. These advantages make In_2_O_3_ a promising sensing material for detecting TEA, ethanol, acetone, NO_2_, H_2_S, and other gases [8,9]. Nevertheless, conventional In_2_O_3_ materials often suffer from particle aggregation, insufficient porosity, and limited active surface sites, which restrict further enhancement of their gas-sensing performance.

Constructing porous nanostructures is an effective strategy to improve the sensing behavior of In_2_O_3_. Porous architectures can provide enlarged gas-accessible surfaces, shorten diffusion pathways, and accelerate the adsorption, reaction, and desorption processes of target gas molecules [10,11]. In recent years, metal–organic frameworks have been widely employed as precursors or templates for preparing porous metal oxides because of their tunable structures, high porosity, and homogeneous metal-ligand distribution [12,13]. Upon thermal decomposition, MOFs can be converted into corresponding metal oxides while retaining or deriving porous frameworks, which is beneficial for gas sensing.

During MOF synthesis, solvent composition plays a crucial role in determining crystal nucleation, growth behavior, and the final porous structure of the derived oxide [14,15]. N,N-dimethylformamide is commonly used as a polar organic solvent to dissolve organic ligands and promote MOF crystal growth, whereas ethanol can modify the polarity, coordination environment, and nucleation rate of the reaction system. Therefore, adjusting the DMF/ethanol ratio provides an effective way to regulate the structure and morphology of MOF precursors, thereby influencing the crystal structure, pore characteristics, oxygen-vacancy concentration, and chemisorbed oxygen content of the derived In_2_O_3_.

In this study, In-based MOF precursors were synthesized using indium nitrate as the metal source and terephthalic acid as the organic ligand by tuning the DMF/ethanol solvent composition. Porous In_2_O_3_ nanostructures assembled from nanoparticles were subsequently obtained through thermal treatment. The effects of the solvent ratio on the crystal structure, morphology, pore structure, surface oxygen species, and TEA-sensing performance of MOF-derived In_2_O_3_ were systematically investigated. The results show that MOF-In_2_O_3_-2, although not possessing the largest specific surface area, exhibited the highest oxygen-vacancy and chemisorbed oxygen contents, leading to the best response, good selectivity, and stable sensing behavior toward TEA at 240 °C.

## 2. Experimental Section

### 2.1. Chemical Reagents

Indium nitrate, terephthalic acid, N,N-dimethylformamide, ethanol, and other reagents used in this work were of analytical grade and purchased from Shandong Yasi Reagent Company. All chemicals were used as received without further purification. Deionized water was used throughout the experiments.

### 2.2. Preparation of MOF-Derived Porous In_2_O_3_

The MOF-derived porous In_2_O_3_ nanostructures were prepared by a solvothermal method followed by thermal treatment. Typically, 3 mmol of indium nitrate was dissolved in a mixed solvent composed of DMF and ethanol under magnetic stirring. Three DMF/ethanol volume ratios, namely 1:3, 1:1, and 3:1, were used to investigate the effect of solvent composition on precursor formation. Subsequently, 3 mmol of terephthalic acid was added into the solution, followed by continuous stirring until a homogeneous precursor solution was obtained. The resulting solution was transferred into a 45 mL Teflon-lined stainless-steel autoclave, sealed, and heated at 80 °C for 24 h. After naturally cooling to room temperature, the precipitate was collected by centrifugation and washed three times with deionized water and ethanol to remove residual solvent and unreacted species. The obtained products prepared with DMF/ethanol volume ratios of 1:3, 1:1, and 3:1 were dried overnight at 60 °C and denoted as MOF-1, MOF-2, and MOF-3, respectively. The dried MOF precursors were then ground thoroughly and calcined in a muffle furnace at 400 °C for 3 h in air. The final oxide products derived from MOF-1, MOF-2, and MOF-3 were denoted as MOF-In_2_O_3_-1, MOF-In_2_O_3_-2, and MOF-In_2_O_3_-3, respectively.

### 2.3. Characterization

The crystal structure of the synthesized nanomaterials was examined by X-ray diffraction (XRD, X’Pert Powder, Panalytical) using Cu Kα radiation (λ = 1.54056 Å), scanned over a 10–90° range at 2°/min. Their microstructure and morphology were investigated via scanning electron microscopy (SEM, Zeiss G500, Carl Zeiss AG) and transmission electron microscopy (TEM, Tecnai G20, Thermo Fisher Scientific). Fourier transform infrared spectroscopy (FTIR, Nicolet iS50, Thermo Fisher Scientific) was used to identify the changes in functional groups after thermal conversion, and UV–Vis absorption spectroscopy (UV-3600iPlus, Shimadzu) was performed to investigate the optical absorption properties. Pore-size distribution and specific surface area were determined using the BJH model and BET method (ASAP 2420, Micromeritics (Shanghai) Instrument Co., Ltd.). Surface elemental composition was analyzed by X-ray photoelectron spectroscopy (XPS, Thermo Scientific™ K-Alpha™+, Thermo Fisher Scientific), while oxygen-vacancy-related defects were further evaluated through electron paramagnetic resonance (EPR, A300, Bruker Magnetic Resonance).

### 2.4. Fabrication and Measurement of Gas Sensors

An appropriate amount of MOF-In_2_O_3_ powder was dispersed in a small volume of deionized water or ethanol and thoroughly ground to yield a homogeneous slurry. The resulting slurry was uniformly coated onto the outer surface of a ceramic tube integrated with pre-fabricated electrodes. After complete drying, a Ni-Cr heating coil was inserted coaxially into the ceramic tube to serve as the heating element. The fabricated sensors were subsequently subjected to an aging treatment prior to gas-sensing characterization.

Gas-sensing experiments were conducted via a gas-sensing measurement system. The operating temperature was regulated by modifying the voltage applied to the heating coil. TEA vapor was introduced into the test chamber by injection and allowed to diffuse to reach the desired concentration. The resistance of the sensor in air was denoted as Ra, and that in the target gas was denoted as Rg. Since In_2_O_3_ is an n-type semiconductor and its resistance decreases upon exposure to reducing TEA, the sensor response was defined as [16,17]:

S = Ra/Rg(1)

The response and recovery times were defined as the time required for the resistance change to reach 90% of the total variation during gas exposure and air recovery, respectively [18,19]. To quantify the selectivity toward TEA, the selectivity coefficient was calculated as K = S_TEA_/S_interferent_, where STEA and Sinterferent represent the responses toward TEA and an interfering gas under the same testing conditions, respectively. Humidity-dependent sensing measurements were also performed toward 100 ppm TEA at 240 °C under different relative humidity conditions.

## 3. Results and Discussion

### 3.1. Formation of MOF-Derived Porous In_2_O_3_ Nanostructures

**Figure 1:**
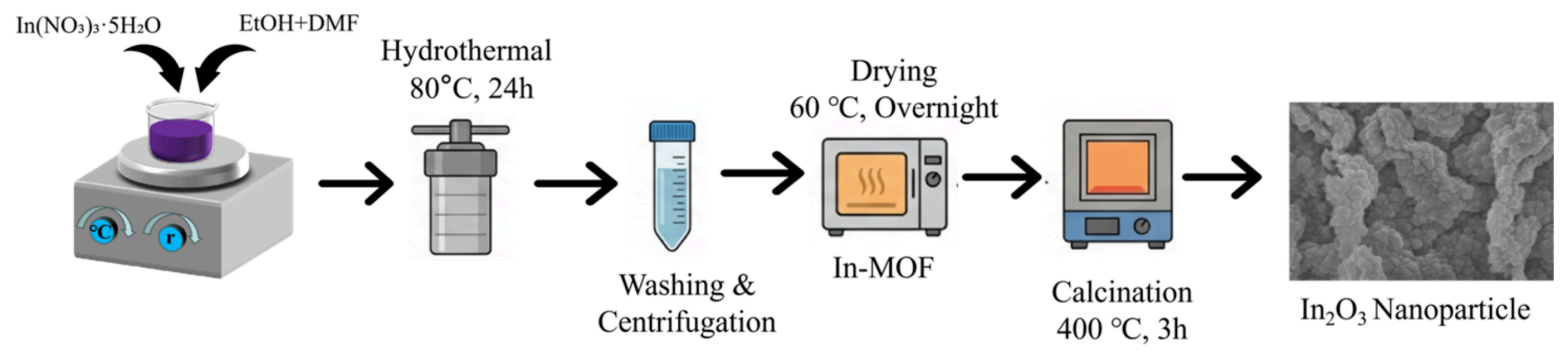
Schematic illustration of the synthesis process of MOF-derived porous In_2_O_3_ structures assembled by nanoparticles.

Figure 1 schematically illustrates the formation process of the MOF-derived porous In_2_O_3_ nanostructures. During the solvothermal reaction, In^3+^ ions coordinate with terephthalic acid ligands to form In-based MOF precursors [20]. The mixed solvent composed of DMF and ethanol not only affects ligand dissolution and the coordination environment of In^3+^, but also regulates the nucleation and growth kinetics of the MOF precursor. In this work, the DMF/ethanol ratios of 1:3, 1:1, and 3:1 produced the precursor samples corresponding to MOF-In_2_O_3_-1, MOF-In_2_O_3_-2, and MOF-In_2_O_3_-3, respectively. The SEM images of the three MOF precursors and the XRD pattern of representative MOF-2 are provided in Figure S1, confirming the formation of In-based MOF precursors before calcination. During subsequent thermal treatment at 400 °C, the organic ligands gradually decompose and release gaseous products, while the In nodes are oxidized and converted into In_2_O_3_. As a result of organic component decomposition and gas release, porous structures assembled from In_2_O_3_ nanoparticles are formed.

**Figure 2:**
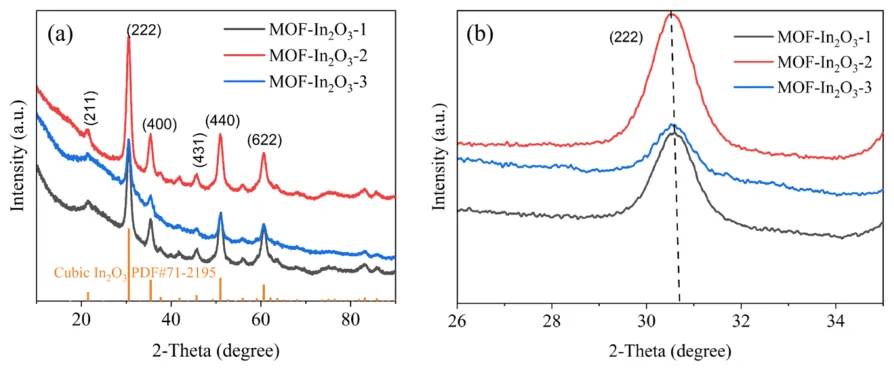
XRD analysis of the prepared samples: (**a**) XRD patterns and (**b**) enlarged view of the (222) diffraction peak.

Figure 2a shows the XRD patterns of the prepared MOF-In_2_O_3_ samples. All diffraction peaks exhibit a high degree of consistency with the standard cubic In_2_O_3_ phase, and no impurity peaks are detected, suggesting that the In-based MOF precursors were fully transformed into pure In_2_O_3_ subsequent to calcination at 400 °C. Figure 2b presents the enlarged view of the main (222) diffraction peak. A slight shift in peak position can be observed among the samples prepared with different solvent ratios. All three samples have a mean crystallite size of ~7.6 nm, indicating that the DMF/ethanol ratio has no significant effect on crystallite growth, as shown in Table 1. The MOF-derived route effectively suppresses excessive crystal growth during calcination. However, the unit-cell volumes differ noticeably. MOF-In_2_O_3_-1, MOF-In_2_O_3_-2, and MOF-In_2_O_3_-3 show cell volumes of 1030.3, 1038.0, and 1035.0 Å^3^, respectively. Among them, MOF-In_2_O_3_-2 exhibits the largest cell volume, while MOF-In_2_O_3_-1 has the smallest one. The enlargement of the unit cell is commonly associated with lattice distortion or the introduction of point defects such as oxygen vacancies. Oxygen vacancies may induce local lattice expansion around neighboring atoms, thereby increasing the lattice parameter and unit-cell volume [21,22]. Therefore, MOF-In_2_O_3_-2 is expected to contain a relatively high concentration of oxygen vacancies, whereas MOF-In_2_O_3_-1 may possess fewer defects. The moderate DMF/ethanol ratio used for MOF-In_2_O_3_-2 may favor the generation of structural defects during MOF precursor formation, part of which are retained in the In_2_O_3_ lattice after calcination. This inference is further supported by the XPS and gas-sensing analyses discussed below.

**Figure 3:**
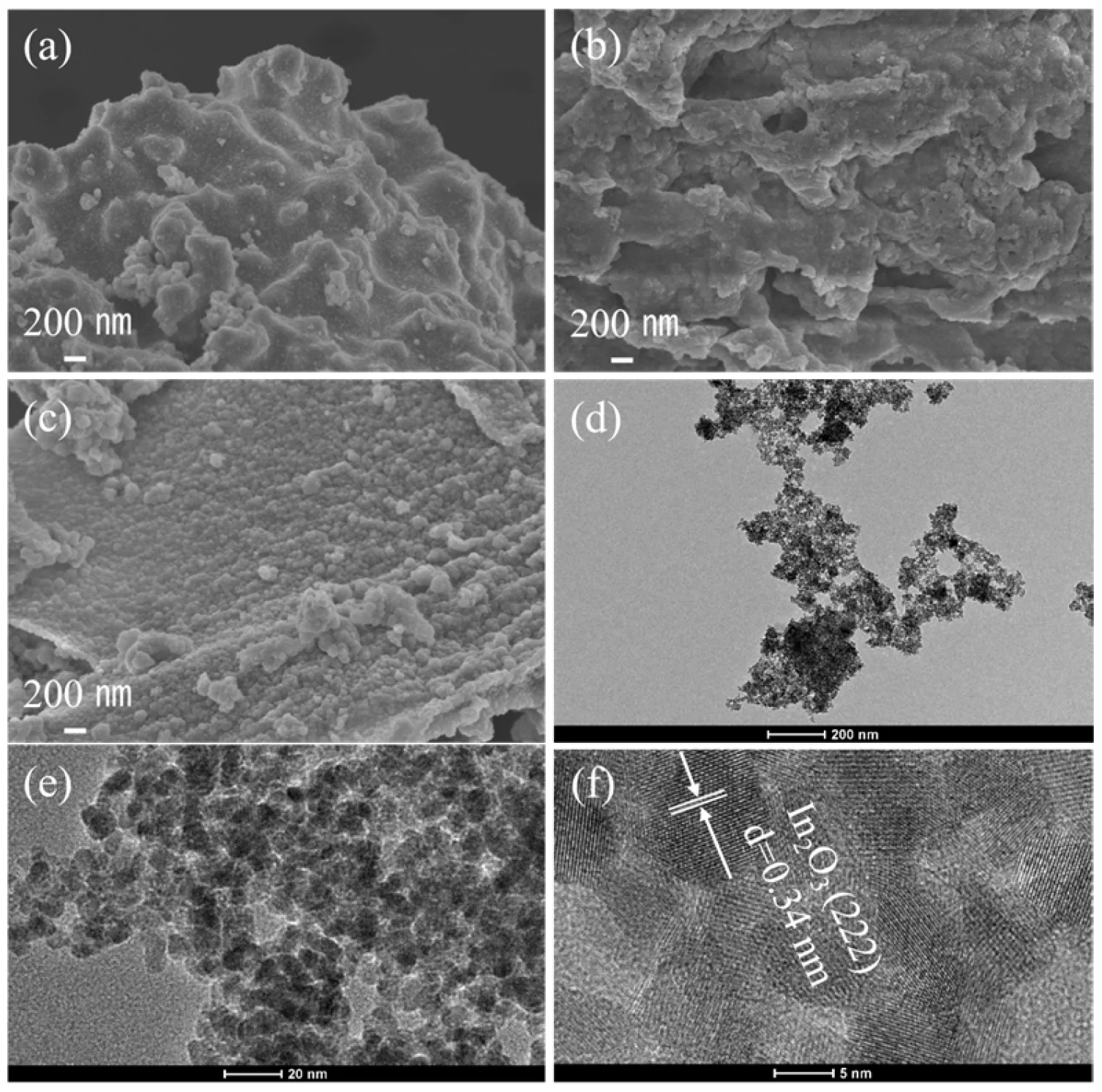
Morphological characterization of the prepared samples: (**a**) MOF-In_2_O_3_-1, (**b**) MOF-In_2_O_3_-2, (**c**) MOF-In_2_O_3_-3, and (**d**–**f**) TEM and HRTEM images of MOF-In_2_O_3_-2.

The SEM, TEM, and HRTEM images of the prepared MOF-In_2_O_3_ samples are shown in Figure 3. The SEM images in Figure 3a–c reveal that all samples exhibit porous structures assembled from nanoparticles. Nevertheless, the particle-packing mode, pore distribution, and structural uniformity vary significantly with the DMF/ethanol ratio. The precursor SEM images in Figure S1 further show that the mixed-solvent composition affects the morphology of the In-MOF precursors before calcination. MOF-In_2_O_3_-1 displays a relatively loose particle assembly with nonuniform pores and some large voids, whereas MOF-In_2_O_3_-2 exhibits a more uniform nanoparticle-assembled porous architecture. Continuous pores with moderate size are formed among adjacent nanoparticles, providing efficient diffusion pathways for gas molecules and abundant interfaces for surface reactions. The TEM image of MOF-In_2_O_3_-2 further confirms that the sample is assembled from numerous small nanoparticles with sizes of approximately 7–8 nm, and abundant mesopores are distributed among the particles. The HRTEM image shows clear lattice fringes with an interplanar spacing of about 0.29 nm, corresponding to the (222) plane of cubic In_2_O_3_, indicating good crystallinity. Overall, the solvent ratio plays a decisive role in regulating the nucleation and growth of the MOF precursor, thereby controlling the morphology and pore structure of the derived In_2_O_3_. The moderate particle-packing density and favorable pore connectivity of MOF-In_2_O_3_-2 provide a structural basis for its enhanced TEA-sensing performance.

**Figure 4:**
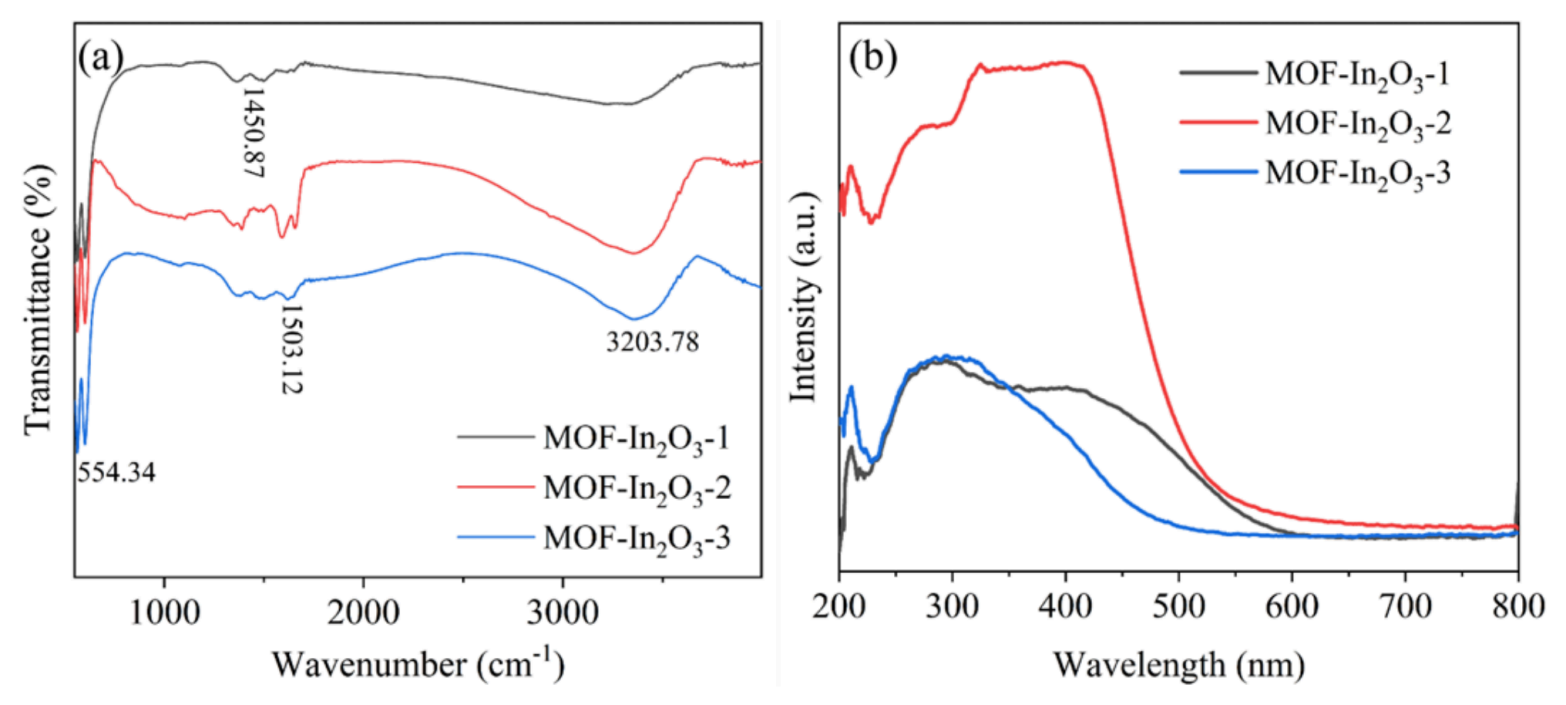
(**a**) FTIR spectra and (**b**) UV–Vis absorption spectra of the prepared samples.

Figure 4 presents the FTIR and UV–Vis absorption spectra of the prepared samples. As shown in Figure 4a, all samples exhibit characteristic absorption bands in the range of approximately 400–600 cm^−1^, which can be assigned to In–O vibrations, confirming the successful formation of In_2_O_3_ [23,24]. Meanwhile, the characteristic bands associated with terephthalic acid, such as those around 1600, 1400, and 1100–1300 cm^−1^, nearly disappear after calcination, indicating the effective decomposition of the organic ligands. A weak residual absorption around 1400 cm^−1^ is still observed for MOF-In_2_O_3_-1, while MOF-In_2_O_3_-2 and MOF-In_2_O_3_-3 show cleaner spectra, suggesting that a higher ethanol proportion may slightly affect the completeness of precursor decomposition. The UV–Vis absorption spectra shown in Figure 4b reveal obvious absorption edges in the range of 300–400 nm for all three samples, corresponding to the intrinsic wide-bandgap absorption of In_2_O_3_ [25]. Notably, the absorption-edge position and intensity vary with the solvent composition. MOF-In_2_O_3_-2 exhibits a slight red shift compared with the other samples, whereas MOF-In_2_O_3_-3 shows a relative blue-shift tendency. Such shifts are closely related to defect-state density, oxygen-vacancy concentration, and lattice distortion in semiconductor materials [26,27]. The introduction of oxygen vacancies can create defect energy levels within the bandgap of In_2_O_3_, thereby reducing the effective bandgap and causing a red shift in the absorption edge. Combined with the XPS results, MOF-In_2_O_3_-2 has the highest oxygen-vacancy proportion of 25.59%, which is consistent with its red-shifted absorption edge. In contrast, MOF-In_2_O_3_-3 has the lowest oxygen-vacancy proportion of 20.91%, in agreement with its relatively blue-shifted absorption behavior. Moreover, MOF-In_2_O_3_-2 shows the strongest absorbance over the measured wavelength range, which may be attributed to its defect-rich surface. These results demonstrate that adjusting the DMF/ethanol ratio can effectively tune the defect structure and electronic band characteristics of In_2_O_3_, with oxygen-vacancy concentration being a key factor affecting its optical absorption behavior.

**Figure 5:**
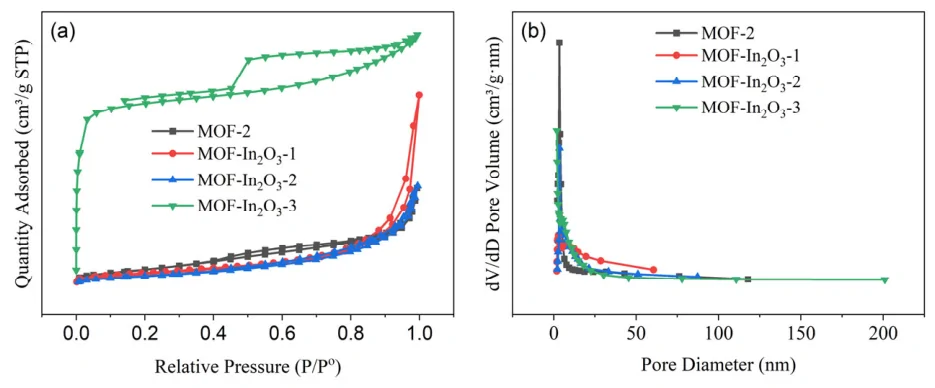
Porous structure analysis of the prepared samples: (**a**) N_2_ adsorption–desorption isotherms and (**b**) pore size distribution curves.

Figure 5 shows the N_2_ adsorption–desorption isotherms and the corresponding pore-size distributions. As listed in Table 2, the MOF-2 precursor exhibits a high specific surface area of 571.80 m^2^/g and an average pore diameter of 4.80 nm, indicating its highly porous framework structure. After calcination, the BET surface areas of MOF-In_2_O_3_-1, MOF-In_2_O_3_-2, and MOF-In_2_O_3_-3 are 75.29, 60.25, and 49.39 m^2^/g, respectively, with corresponding average pore diameters of 7.85, 19.85, and 10.70 nm. These results indicate that the derived In_2_O_3_ samples possess mesoporous structures with moderate surface areas. Although MOF-In_2_O_3_-2 does not exhibit the largest specific surface area among the calcined samples, it possesses the largest average pore diameter, which is favorable for the diffusion and transport of relatively large TEA molecules. Therefore, the TEA-sensing performance is not governed solely by specific surface area; pore accessibility, surface oxygen species, and defect-related active sites should be considered together.

**Figure 6:**
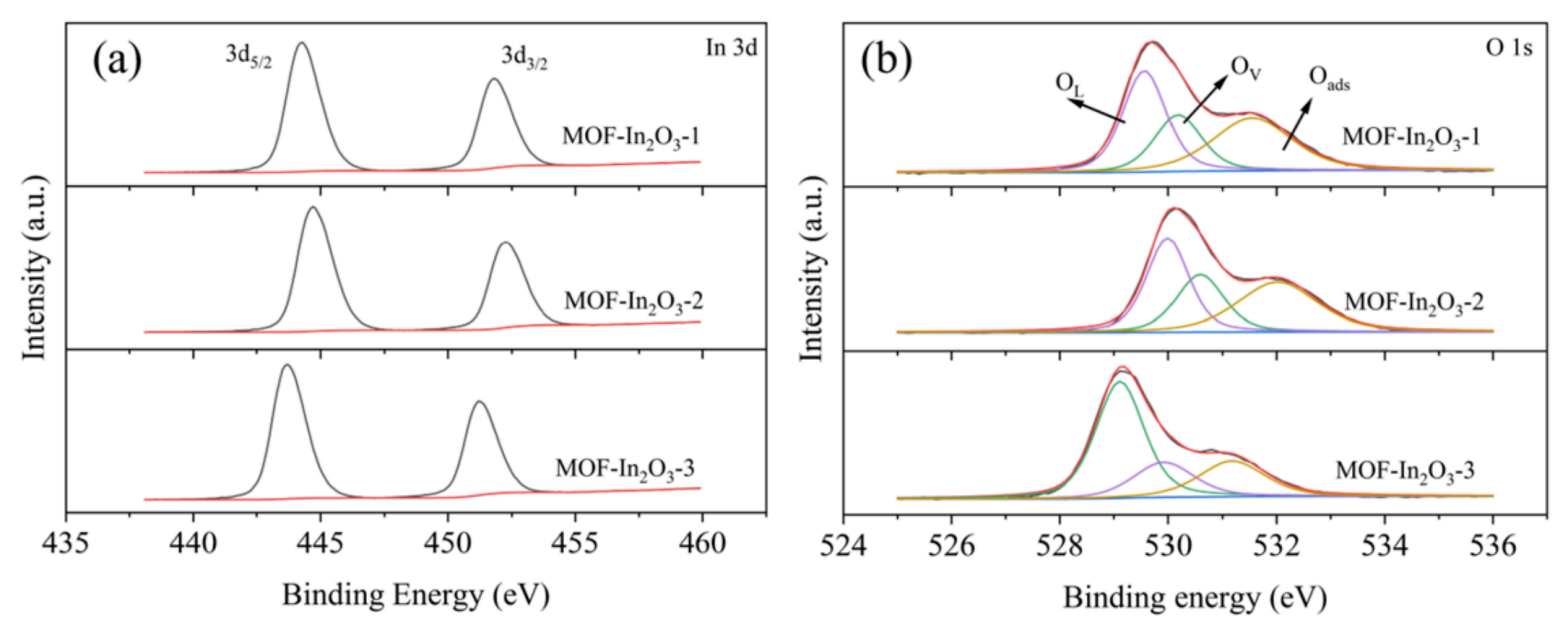
XPS analysis of the prepared samples: (**a**) In 3d and (**b**) O 1s spectra.

Figure 6a shows the high-resolution In 3d XPS spectra of the prepared samples. Two characteristic peaks located at approximately 444.5 and 452.1 eV are observed, corresponding to In 3d_5_/_2_ and In 3d_3_/_2_ of In^3+^ in In_2_O_3_, respectively [28,29]. The O 1s spectra shown in Figure 6b can be deconvoluted into three components: lattice oxygen (O_L_, approximately 529.8 eV), oxygen species related to oxygen vacancies (O_V_, approximately 531.0 eV), and surface chemisorbed oxygen species (O_ads_, approximately 532.2 eV) [30,31]. The relative proportions are summarized in Table 3. The OV contents of MOF-In_2_O_3_-1, MOF-In_2_O_3_-2, and MOF-In_2_O_3_-3 are 23.64%, 25.59%, and 20.91%, respectively, while the corresponding Oads contents are 37.30%, 37.21%, and 23.14%. MOF-In_2_O_3_-2 exhibits the highest oxygen-vacancy-related component while maintaining a high chemisorbed oxygen content [32]. Moreover, the EPR spectra in Figure S2 show an obvious oxygen-vacancy-related signal, and MOF-In_2_O_3_-2 displays the strongest EPR response among the three samples, which is consistent with the XPS O 1s analysis. These results suggest that the 1:1 DMF/ethanol ratio is more favorable for generating defect-related active sites and surface oxygen species in the derived In_2_O_3_.

### 3.2. TEA Gas-Sensing Performance

**Figure 7:**
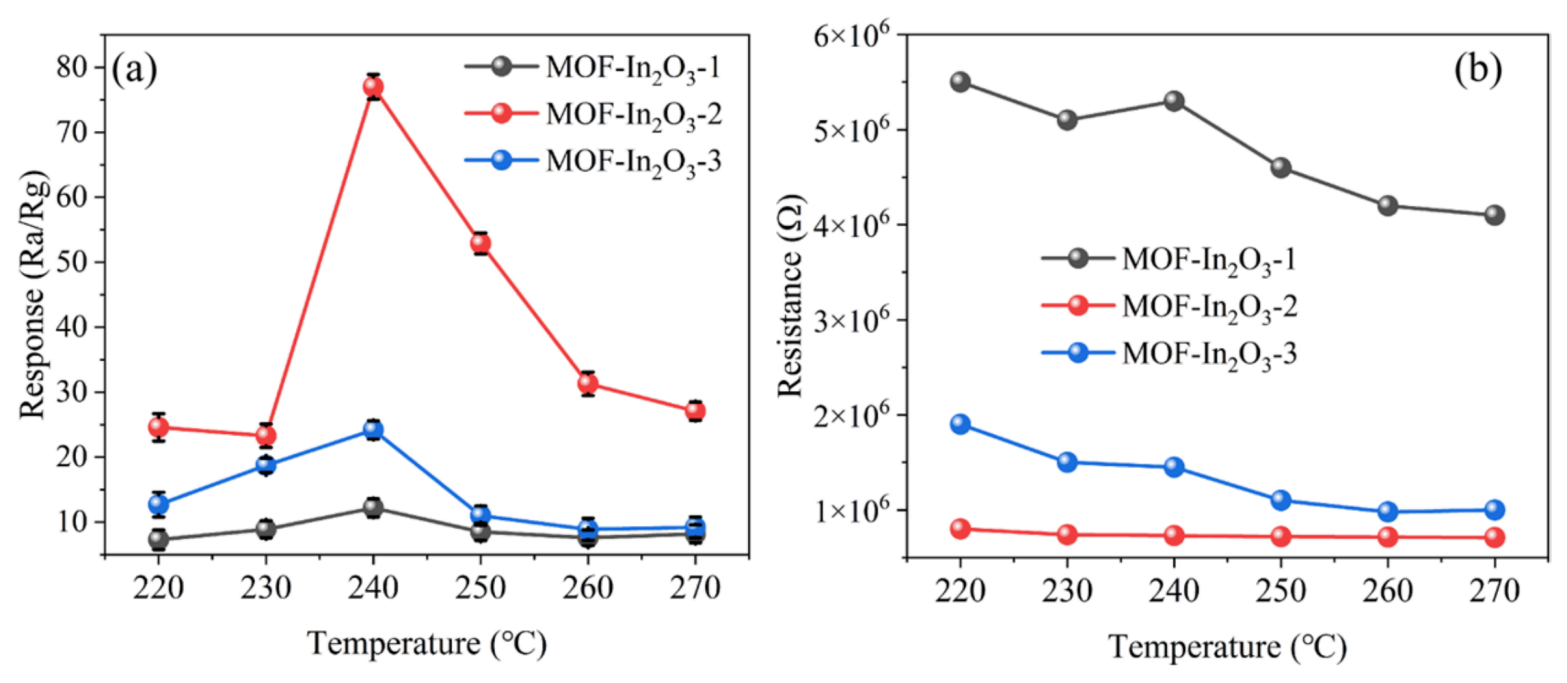
Temperature-dependent sensing properties of the prepared samples: (**a**) responses toward 100 ppm TEA at different operating temperatures and (**b**) resistance variations at different operating temperatures.

As shown in Figure 7a, the responses of all samples first increase and then decrease with increasing temperature, reaching the maximum at 240 °C. The error bars represent the standard deviations of repeated measurements. Therefore, 240 °C is identified as the optimal operating temperature for this sensing system. At relatively low temperatures, TEA molecules are insufficiently activated, and the reaction between surface adsorbed oxygen and target gas molecules proceeds slowly, resulting in a weak response. As temperature rises to 240 °C, TEA molecules gain sufficient activation energy, accelerating surface catalytic oxidation and markedly enhancing the response. Above 280 °C, faster gas desorption, reduced adsorbed-oxygen stability, and shorter effective reaction time diminish the response. Among all samples, MOF-In_2_O_3_-2 exhibits the highest response at every tested temperature. In particular, its response toward 100 ppm TEA reaches 77 at 240 °C, which is much higher than those of MOF-In_2_O_3_-1 and MOF-In_2_O_3_-3. Figure 7b shows that the resistance of all samples decreases with increasing temperature, consistent with the negative temperature coefficient behavior of semiconductor materials. Notably, MOF-In_2_O_3_-2 maintains a moderate resistance over the tested temperature range, which is beneficial for signal acquisition and signal-to-noise optimization. Therefore, 240 °C was selected as the operating temperature for subsequent sensing measurements.

**Figure 8:**
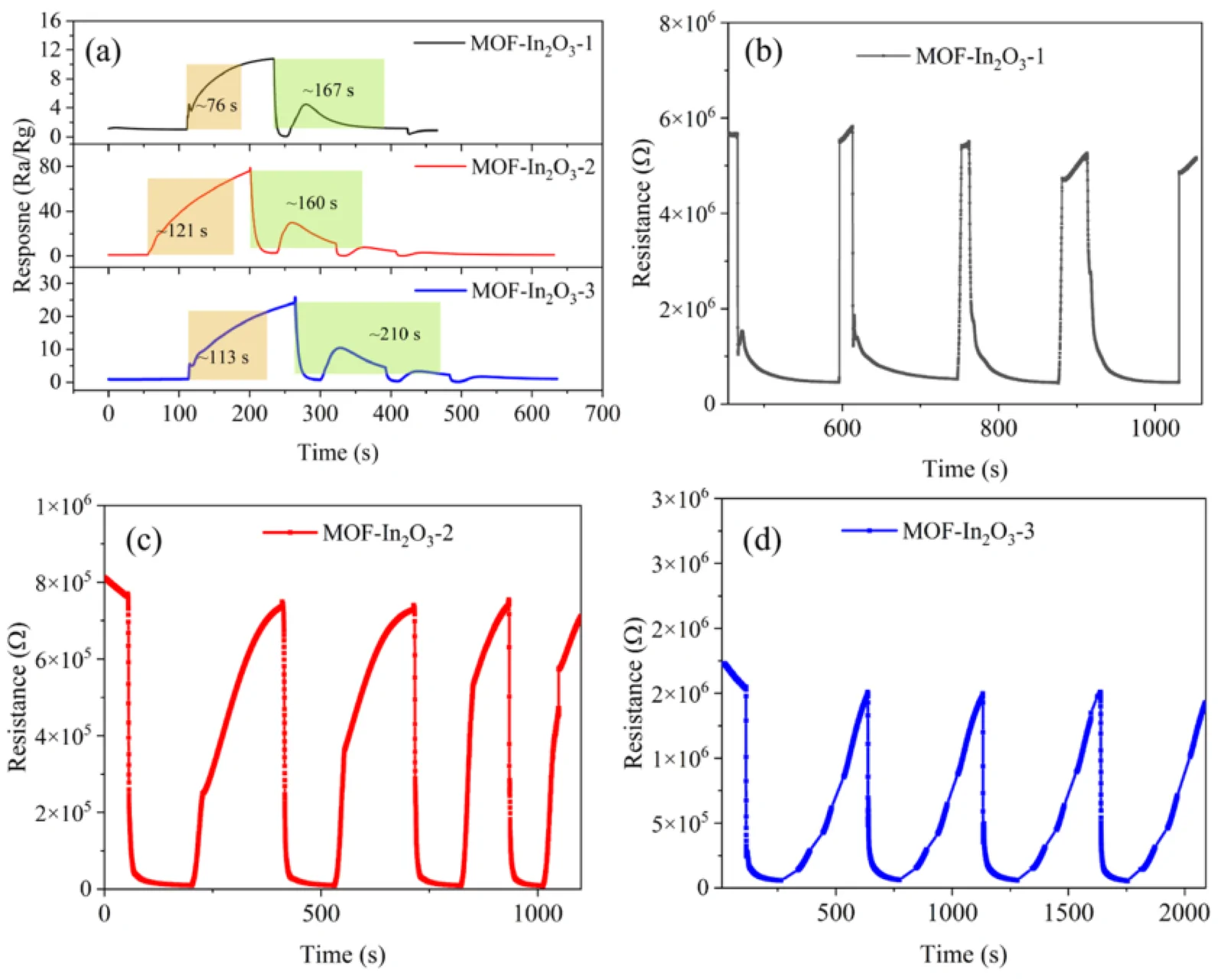
Dynamic sensing characteristics of the prepared samples toward 100 ppm TEA at 240 °C: (**a**) response/recovery curves and dynamic response curves of (**b**) MOF-In_2_O_3_-1, (**c**) MOF-In_2_O_3_-2, and (**d**) MOF-In_2_O_3_-3.

Figure 8b–d display the dynamic response curves of MOF-In_2_O_3_-1, MOF-In_2_O_3_-2, and MOF-In_2_O_3_-3, respectively. When exposed to TEA, the sensor resistance decreases rapidly; after the gas atmosphere is switched back to air, the resistance gradually recovers to its initial value. This reversible resistance variation indicates that all samples exhibit stable sensing behavior toward TEA. MOF-In_2_O_3_-2 shows the largest response amplitude, with a response value of 77 toward 100 ppm TEA. Its response and recovery times are 121 and 160 s, respectively. By comparison, MOF-In_2_O_3_-1 and MOF-In_2_O_3_-3 show response values of 45 and 36, respectively. The excellent dynamic response of MOF-In_2_O_3_-2 can be attributed to the synergistic effect between its porous structure and surface chemistry. SEM and TEM observations demonstrate that MOF-In_2_O_3_-2 possesses a uniform nanoparticle-assembled porous architecture, which provides efficient channels for gas diffusion. XPS and EPR analyses further indicate that it contains abundant oxygen-vacancy-related defects and a high proportion of chemisorbed oxygen species. Therefore, the superior sensing behavior of MOF-In_2_O_3_-2 should be mainly related to its balanced pore accessibility and surface-active oxygen species rather than to a simple surface-area effect.

**Figure 9:**
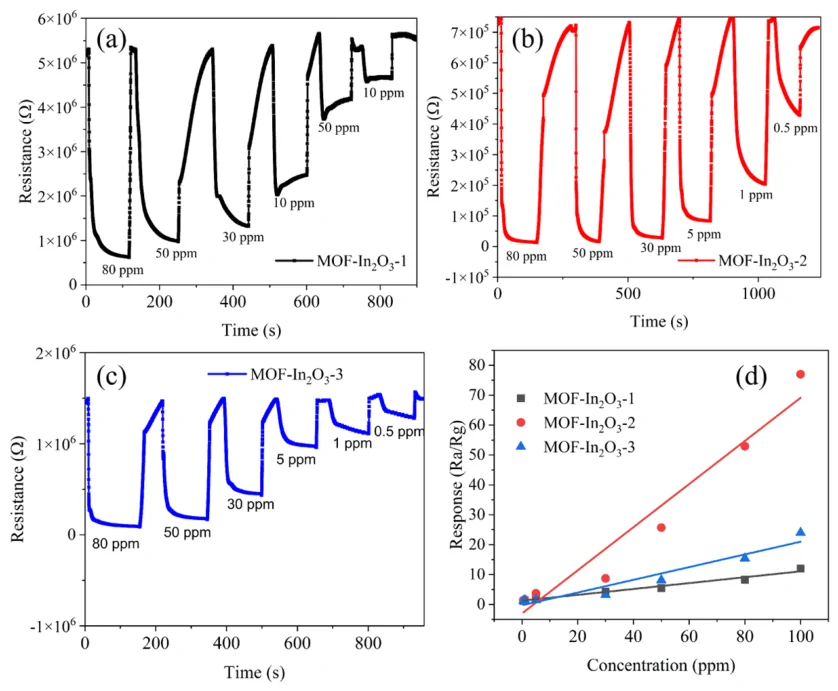
Concentration-dependent sensing properties of the prepared samples toward TEA at 240 °C: (**a**–**c**) dynamic response curves and (**d**) relationship between response and TEA concentration.

Figure 9a–c show the dynamic response curves of the prepared sensors toward different concentrations of TEA ranging from 0.5 to 100 ppm at 240 °C. Figure 9d presents the relationship between sensor response and TEA concentration. With increasing TEA concentration from 0.5 to 100 ppm, the responses of all sensors increase monotonically, indicating that the MOF-In_2_O_3_ sensors can effectively detect TEA over a broad concentration range. In the low-concentration region below 10 ppm, the response increases rapidly with increasing concentration because a small number of TEA molecules can react with surface adsorbed oxygen and induce a noticeable resistance change. At higher concentrations, the response gradually tends to increase more slowly, which may be due to the gradual saturation of surface active sites. MOF-In_2_O_3_-2 exhibits the highest response at all tested concentrations. Even at 0.5 ppm, it still produces a distinguishable response signal, indicating that its detection limit can reach 0.5 ppm. In contrast, MOF-In_2_O_3_-3 shows a relatively weak response at low concentrations, which is consistent with its lower oxygen-vacancy and chemisorbed oxygen contents. The response–concentration curves further confirm that MOF-In_2_O_3_-2 outperforms the other two samples throughout the entire concentration range and exhibits a good positive correlation between response and TEA concentration. This concentration-dependent behavior suggests that MOF-In_2_O_3_-2 is suitable for quantitative detection of TEA from trace to relatively high concentrations.

**Figure 10:**
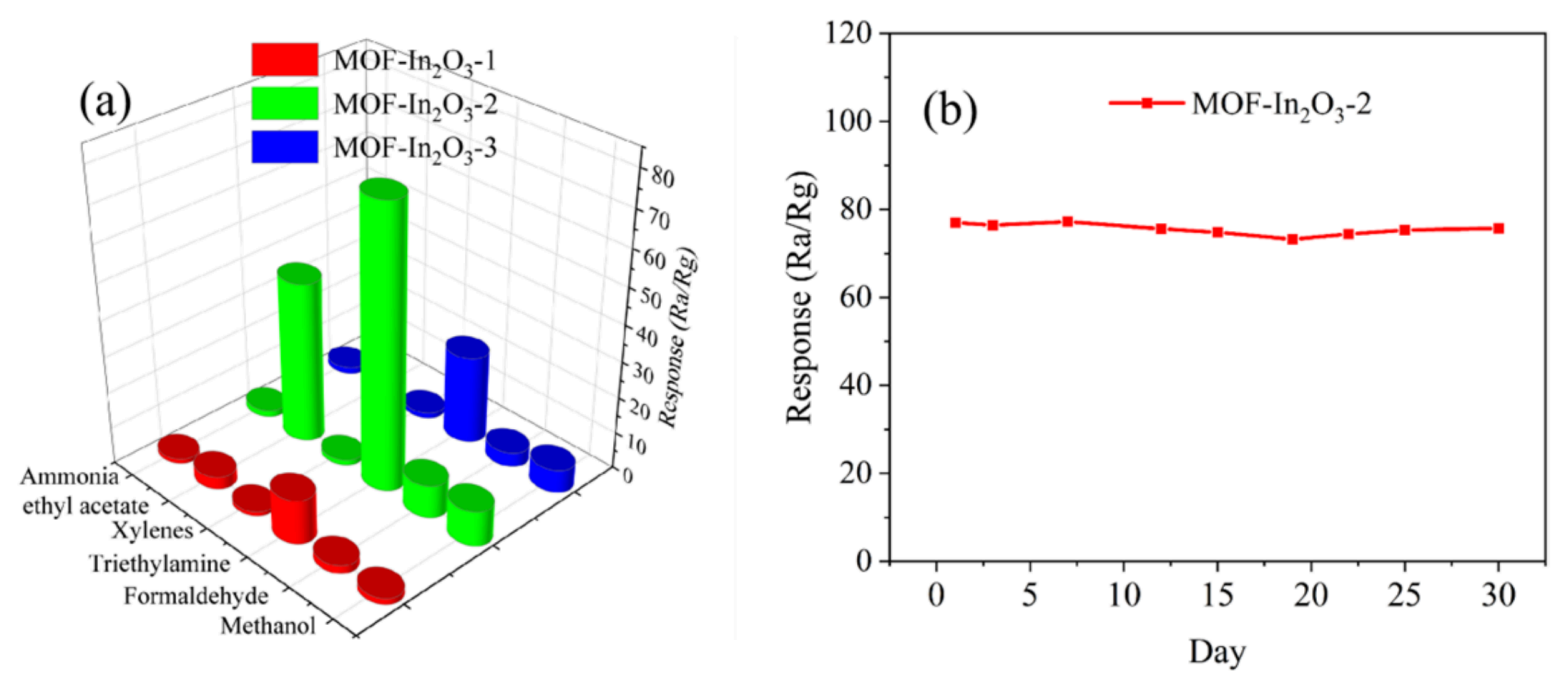
Selectivity and stability of the prepared samples: (**a**) responses toward 100 ppm of various gases at 240 °C and (**b**) 30-day stability of MOF-In_2_O_3_-2 toward 100 ppm TEA.

Figure 10a compares the responses of the prepared sensors toward 100 ppm of different gases, including TEA, ammonia, ethyl acetate, xylenes, formaldehyde, and methanol, at 240 °C. MOF-In_2_O_3_-2 shows a response of 77 toward TEA, which is much higher than its responses toward the interfering gases. To quantitatively evaluate selectivity, the selectivity coefficient was calculated as K = S_TEA_/S_interferent_. Since the responses of MOF-In_2_O_3_-2 toward the interfering gases are all lower than 10, the selectivity coefficients of TEA relative to these interfering gases are higher than 7.7, confirming the preferential response of MOF-In_2_O_3_-2 toward TEA. This high TEA selectivity arises from TEA’s relatively low ionization energy, strong electron-donating capability, and the interaction between the nitrogen lone electron pair and Lewis acidic surface In^3+^ sites [33,34]. Figure 10b shows the response variation in the MOF-In_2_O_3_-2 sensor toward 100 ppm TEA over 30 days, confirming good long-term stability. In addition, the humidity-dependent results shown in Figure S3 reveal that the response gradually decreases with increasing relative humidity but remains distinguishable even under high-humidity conditions, demonstrating the practical potential of the sensor in humid atmospheres.

Table 4 compares the sensing performance of the MOF-In_2_O_3_-2 sensor with representative In_2_O_3_-based TEA sensors reported in the literature. Some materials, such as ZnO/In_2_O_3_ composite nanofibers or In_2_O_3_ nanocubes, show faster response times or lower detection limits. However, these materials often involve noble-metal modification or relatively complex multistep synthesis procedures. In contrast, the MOF-In_2_O_3_-2 sensor developed in this work is prepared through a simple mixed-solvent-regulated MOF precursor strategy without noble-metal decoration. It achieves a high response of 77 toward 100 ppm TEA at 240 °C, with a detection limit of 0.5 ppm. Although its response/recovery time of 121/160 s is not the fastest among reported sensors, it remains acceptable for practical TEA detection. The recovery process may be affected by relatively strong adsorption of TEA on oxygen-vacancy-related sites and delayed desorption from porous channels. Overall, MOF-In_2_O_3_-2 shows balanced advantages in high response, good selectivity, low detection limit, long-term stability, humidity tolerance, and facile preparation.

### 3.3. Gas-Sensing Mechanism

In_2_O_3_ is a typical n-type semiconductor, and its gas-sensing behavior mainly relies on surface oxygen adsorption and redox reactions with target gas molecules. In ambient air, oxygen molecules adsorb onto the In_2_O_3_ surface and withdraw electrons from the conduction band, thereby forming adsorbed oxygen species such as O_2_^−^, O^−^, and O^2−^. This interaction widens the surface electron depletion layer and increases the resistance. At the optimal operating temperature of 240 °C, O^−^ is generally considered the dominant adsorbed oxygen species. When the sensor is exposed to reducing TEA gas, TEA molecules react with the surface adsorbed oxygen species and release electrons back to the conduction band of In_2_O_3_, leading to a thinner depletion layer and decreased resistance [1,5]. Since no direct product analysis was performed in this work, the following equations are presented as a proposed sensing pathway:


(2) $$O_{2}(\mathrm{gas})\to O_{2}\left(\mathrm{ads}\right)$$

(3) $$O_{2}\left(\mathrm{ads}\right)+e^{-}\to O_{2}^{-}\left(\mathrm{ads}\right)\;\;\;\;\;\;\left(T\leq 100\;{}^{\circ}C\right)$$

(4) $$O_{2}^{-}\left(\mathrm{ads}\right)+2e^{-}\to 2O^{-}(\mathrm{ads})\;\;\;\;\;\left(100<T\leq 300\;{}^{\circ}C\right)$$

(5) $$O^{-}\left(\mathrm{ads}\right)+e^{-}\to O^{2-}\left(\mathrm{ads}\right)\;\;\;\left(T>300\;{}^{\circ}C\right)$$

The superior TEA-sensing performance of MOF-In_2_O_3_-2 originates from the synergistic contribution of its porous architecture, surface defects, and active oxygen species [45,46]. First, SEM and TEM observations show that MOF-In_2_O_3_-2 consists of uniformly assembled In_2_O_3_ nanoparticles with sizes of approximately 7-8 nm, forming a porous network with the largest average pore diameter of 19.85 nm among the calcined samples. This relatively open pore structure is favorable for the diffusion and transport of TEA molecules and ensures sufficient gas–solid contact interfaces. Second, XPS analysis reveals that MOF-In_2_O_3_-2 has the highest oxygen-vacancy-related O 1s component of 25.59% and a high chemisorbed oxygen proportion of 37.21%. The EPR spectra in Figure S2 further support the presence of oxygen-vacancy-related defects, with MOF-In_2_O_3_-2 showing the strongest signal. Oxygen vacancies can serve as preferential adsorption and activation centers for oxygen molecules, promoting the formation of reactive oxygen species. Chemisorbed oxygen directly participates in TEA oxidation, enabling electron release and a stronger resistance change. In comparison, MOF-In_2_O_3_-3 exhibits a lower BET surface area of 49.39 m^2^/g and a smaller average pore diameter of 10.70 nm than MOF-In_2_O_3_-2. Its lower oxygen-vacancy-related and chemisorbed oxygen contents, at 20.91% and 23.14%, respectively, further limit the number of effective active sites. Therefore, the moderate DMF/ethanol ratio of 1:1 not only constructs a relatively open pore system favorable for TEA diffusion but also enriches surface active oxygen species. The synergistic structural and chemical merits enable MOF-In_2_O_3_-2 to attain highly sensitive and selective detection of TEA at a comparatively low operating temperature.

## 4. Conclusions

In this work, porous In_2_O_3_ nanostructures were synthesized via a mixed-solvent-regulated MOF-derived route, and the influence of the DMF/ethanol ratio on precursor formation, microstructure, surface defect chemistry, and TEA-sensing properties was systematically investigated. Three DMF/ethanol volume ratios of 1:3, 1:1, and 3:1 were employed to prepare In-MOF precursors, which were subsequently converted into MOF-In_2_O_3_-1, MOF-In_2_O_3_-2, and MOF-In_2_O_3_-3 after calcination, respectively. The formation of In-MOF precursors was supported by the precursor SEM images and the representative XRD pattern of MOF-2. BET analysis showed that the MOF-2 precursor possessed a high specific surface area of 571.80 m^2^ g^−1^, while the calcined MOF-In_2_O_3_-2 sample exhibited a relatively open mesoporous structure with the largest average pore diameter of 19.85 nm among the derived oxides. EPR and XPS results further indicated that MOF-In_2_O_3_-2 contained abundant oxygen-vacancy-related defects and chemisorbed oxygen species. Benefiting from the synergistic effect of accessible porous channels, defect-related active sites, and surface active oxygen species, MOF-In_2_O_3_-2 exhibited the best TEA-sensing performance at 240 °C, including a response of 77 toward 100 ppm TEA, response/recovery times of 121/160 s, a detection limit of 0.5 ppm, good stability, and a selectivity coefficient higher than 7.7 relative to the tested interfering gases. Moreover, the sensor maintained a distinguishable response under humid conditions, demonstrating the potential of mixed-solvent-regulated MOF-derived In_2_O_3_ for selective TEA detection.

## Figures and Tables

**Table 1:** Crystal parameters of the prepared samples.

Samples	Grain Size (nm)	Cell Volume (Å^3^)
MOF-In_2_O_3_-1	7.6	1030.3
MOF-In_2_O_3_-2	7.6	1038
MOF-In_2_O_3_-3	7.6	1035

**Table 2:** Specific surface area and average pore diameter of the prepared samples.

Samples	BET Surface Area (m^2^/g)	Average Pore Diameter (nm)
MOF-2	571.80	4.80
MOF-In_2_O_3_-1	75.29	7.85
MOF-In_2_O_3_-2	60.25	19.85
MOF-In_2_O_3_-3	49.39	10.70

**Table 3:** Relative contents of different oxygen species obtained from XPS analysis.

Samples	O_L_ (%)	O_V_ (%)	O_ads_ (%)
MOF-In_2_O_3_-1	39.07	23.64	37.30
MOF-In_2_O_3_-2	37.20	25.59	37.21
MOF-In_2_O_3_-3	55.95	20.91	23.14

**Table 4:** Comparison of TEA-sensing performance between the present sensor and previously reported materials.

Materials	Method	T (°C)	Concentration (ppm)	S	Response/Recovery Time (s)	LOD (ppm)	Ref
ZnO/In_2_O_3_ nanofibers	MOF	250	50	35	3/880	0.1	[35]
Cubic-like In_2_O_3_	solvothermal	250	100	60.26	114/210	0.89	[36]
Au-In_2_O_3_ microspheres	noble metal modified	360	5	~1.5	30/>60	0.2	[37]
Pd-In_2_O_3_	noble metal modified	240	50	18	4/17	1	[38]
Porous In_2_O_3_ nanoplates	facile solvothermal	320	50	7.8	10/19	2	[39]
In_2_O_3_ nanocubes	MOF	350	100	~21.6	1/28	0.1	[40]
In_2_O_3_ microtube	MOF	140	1	145	5/20	0.1	[41]
MOF-In_2_O_3_	MOF	200	10	~7	2/78	0.5	[42]
porous In_2_O_3_ flower	MOF	140	20	35.2	2/222	0.3	[43]
In_2_O_3_-NiO Composites	MOF	200	100	33.9	140/68	0.5	[44]

## Data Availability

The original contributions presented in this study are included in the article. Further inquiries can be directed to the corresponding author.

## Supplementary Materials

The following supporting information can be downloaded at: https://www.mdpi.com/article/10.3390/ma19163442/s1, Figure S1: Morphological characterization of the prepared samples: (a) MOF-1, (b) MOF-2, (c) MOF-3, and (d) XRD analysis of the prepared MOF-2 samples; Figure S2: The EPR patterns of the prepared samples; Figure S3: The response of the MOF-In_2_O_3_-2 sensor to 100 ppm TEA at 240 ℃ under different humidity conditions.
