# Supplementary material for: Mixed-Solvent-Regulated MOF-Derived Porous In2O3 Nanostructures for Enhanced Triethylamine Gas Sensing
Source: Materials (Basel). 2026 Aug 13;19(16):3442. doi: 10.3390/ma19163442 (PMC13514372; doi:10.3390/ma19163442)
Supplement: Supplementary file 1 [file materials-19-03442-s001.zip › materials-4452899-supplementary.pdf]

Mixed-Solvent-Regulated MOF-Derived Porous In_2O_3 Nanostructures for Enhanced Triethylamine Gas Sensing

Shuhao Shen, Jing Li, Rui Fang, Yongli Zhu, Wenbo Qin *

School of Information Engineering, Suzhou University, Suzhou 234000, China

*   Corresponding author: Wenbo Qin (qwb_suzhou@163.com)

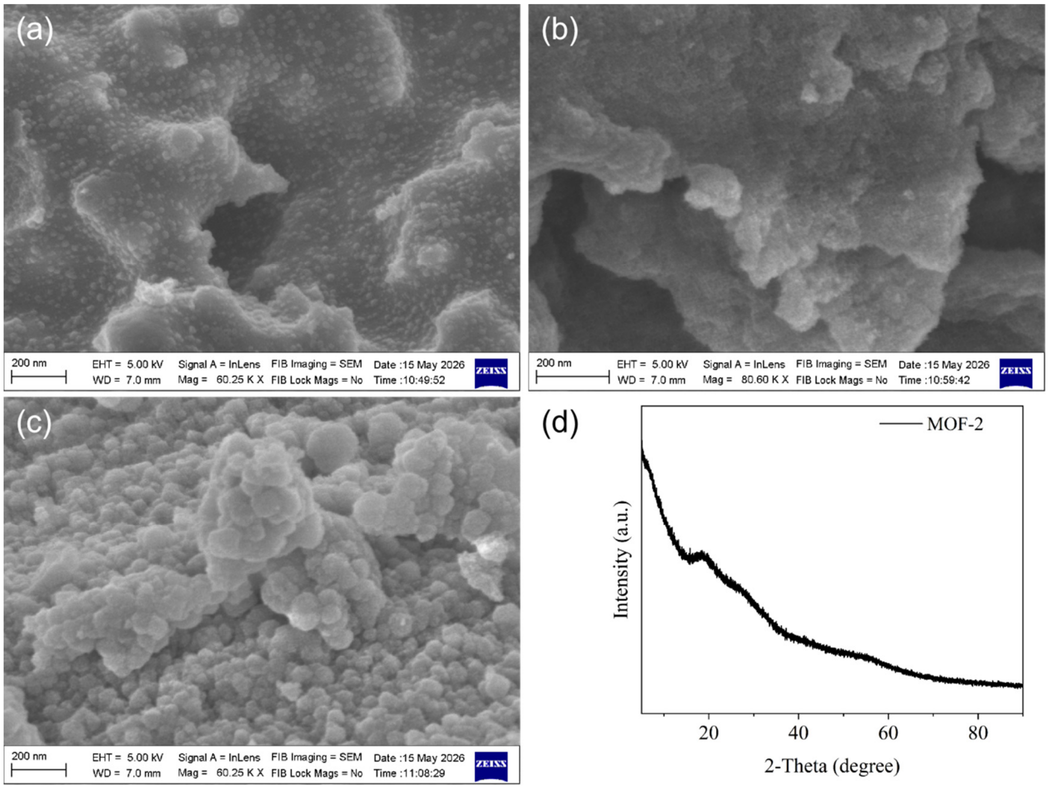

Figure S1 Morphological characterization of the prepared samples: (a) MOF-1, (b) MOF-2, (c) MOF-3, and (d) XRD analysis of the prepared MOF-2 samples.

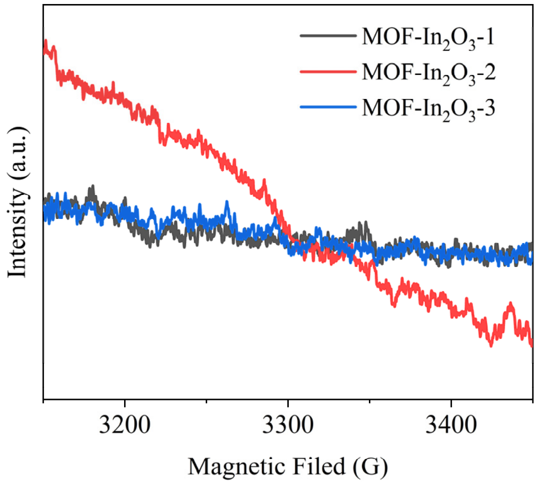

Figure S2 The EPR patterns of the prepared samples.

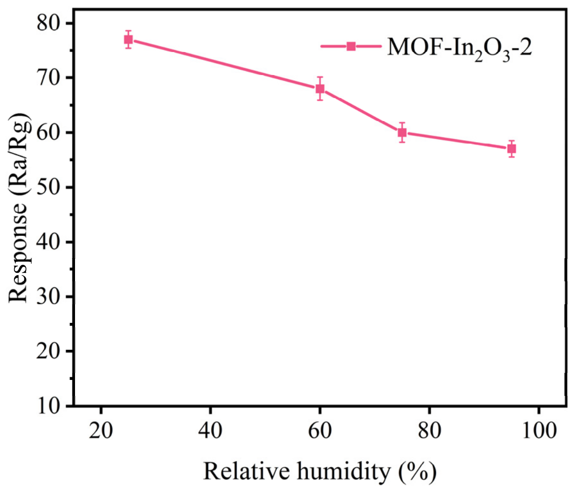

Figure S3 The response of the MOF-In_2O_3-2 sensor to 100 ppm TEA at 240℃ under different humidity conditions.
